# Aberrant MicroRNA Expression and Its Implications for Uveal Melanoma Metastasis

**DOI:** 10.3390/cancers11060815

**Published:** 2019-06-12

**Authors:** Kyra N. Smit, Jiang Chang, Kasper Derks, Jolanda Vaarwater, Tom Brands, Rob M. Verdijk, Erik A.C. Wiemer, Hanneke W. Mensink, Joris Pothof, Annelies de Klein, Emine Kilic

**Affiliations:** 1 Department of Ophthalmology, Erasmus University MC, 3015 GD Rotterdam, The Netherlands; k.n.smit@erasmusmc.nl (K.N.S.); j.vaarwater@erasmusmc.nl (J.V.); 2 Department of Clinical Genetics, Erasmus University MC, 3015 GD Rotterdam, The Netherlands; t.brands@erasmusmc.nl (T.B.); a.deklein@erasmusmc.nl (A.d.K.); 3 Department of Molecular Genetics, Erasmus University MC, 3015 GD Rotterdam, The Netherlands; j.chang@erasmusmc.nl (J.C.); kasper.derks@mumc.nl (K.D.); j.pothof@erasmusmc.nl (J.P.); 4 Department of Pathology, Section Ophthalmic Pathology, Erasmus University MC, 3015 GD Rotterdam, The Netherlands; r.verdijk@erasmusmc.nl; 5 The Rotterdam Eye Hospital, 3011 BH Rotterdam, The Netherlands; h.mensink@oogziekenhuis.nl; 6 Department of Medical Oncology, Erasmus University MC, 3015 GD Rotterdam, The Netherlands; e.wiemer@erasmusmc.nl

**Keywords:** uveal melanoma, metastasis, microRNAs, mRNA expression, IPA pathway analysis

## Abstract

Uveal melanoma (UM) is the most frequently found primary intra-ocular tumor in adults. It is a highly aggressive cancer that causes metastasis-related mortality in up to half of the patients. Many independent studies have reported somatic genetic changes associated with high metastatic risk, such as monosomy of chromosome 3 and mutations in *BAP1*. Still, the mechanisms that drive metastatic spread are largely unknown. This study aimed to elucidate the potential role of microRNAs in the metastasis of UM. Using a next-generation sequencing approach in 26 UM samples we identified thirteen differentially expressed microRNAs between high-risk UM and low/intermediate-risk UM, including the known oncomirs microRNA-17-5p, microRNA-21-5p, and miR-151a-3p. Integration of the differentially expressed microRNAs with expression data of predicted target genes revealed 106 genes likely to be affected by aberrant microRNA expression. These genes were involved in pathways such as cell cycle regulation, EGF signaling and EIF2 signaling. Our findings demonstrate that aberrant microRNA expression in UM may affect the expression of genes in a variety of cancer-related pathways. This implies that some microRNAs can be responsible for UM metastasis and are promising potential targets for future treatment.

## 1. Introduction

Uveal melanoma (UM) is an aggressive cancer that arises from melanocytes located in the uveal tract of the eye. Although treatment of primary tumors has a high success rate, up to half of the patients develop metastasis which often results in death within several months [1]. UM display chromosomal aberrations and genetic abnormalities that underlie both the development and metastasis of UM tumors. Most tumors carry a *GNAQ* or *GNA11* mutation. These mutations are considered to be tumor-initiating mutations and do not increase the risk of metastasis [2,3,4]. UM patients can be stratified into three different metastatic risk groups; those who have a low-, intermediate-, or high-risk of developing metastasis [5].

High-risk UM harbor a mutation in the tumor suppressor gene BRCA-associated protein (*BAP1*), located on chromosome 3 [6]. Mutations in this gene often coincide with monosomy 3, resulting in loss of expression of the BAP1 protein. BAP1 is a deubiquitinating enzyme known to be active in several cellular processes such as DNA damage response, apoptosis, and chromatin remodeling [7,8,9]. Intermediate-risk tumors carry a mutation in the gene-encoding splicing factor 3 subunit 1 (*SF3B1*), which is part of a protein complex involved in pre-mRNA splicing [5,10,11]. *SF3B1* mutations in UM are known to result in aberrantly spliced transcripts that can either be degraded by nonsense-mediated decay or translated into unique, aberrant proteins. Low-risk UM often harbor a mutation in the eukaryotic translation initiation factor 1A (*EIF1AX*) gene, which is involved in the transfer of methionyl initiator tRNA to the small ribosomal subunit during translation [10].

Besides the classification of UM into different metastatic risk groups based on gene mutations, the disease can also be separated into two subclasses based on mRNA expression analysis. Each subclass has a distinct gene expression profile. Class 2 tumors, which include high-risk UM, have a stem cell-like expression pattern; whereas class 1 tumors, which include low- and intermediate-risk UM, have the transcriptome of a differentiated melanocyte [12].

Another mechanism that is thought to be essential in the development and metastatic progression of a tumor is aberrant expression of microRNAs. MicroRNAs (miRNAs) are small, single-stranded, non-coding RNAs that can regulate gene expression by binding to mRNA [13,14]. Although limited studies of miRNA expression in UM have been done [15,16,17,18,19,20,21,22,23,24], the miRNA profiles of the three risk groups and the downstream effects of any aberrant miRNA expression remains unclear. In this study we; therefore, performed small RNA sequencing in UM tissue. Additionally, mRNA sequencing of all UM samples allowed us to determine associations between miRNAs that were differentially expressed and the expression of their putative downstream mRNA-targets. Our aim was to identify miRNAs that might contribute to the invasive and metastatic potential of UM.

## 2. Results

### 2.1. Sample Collection and Analysis

**Figure 1:**
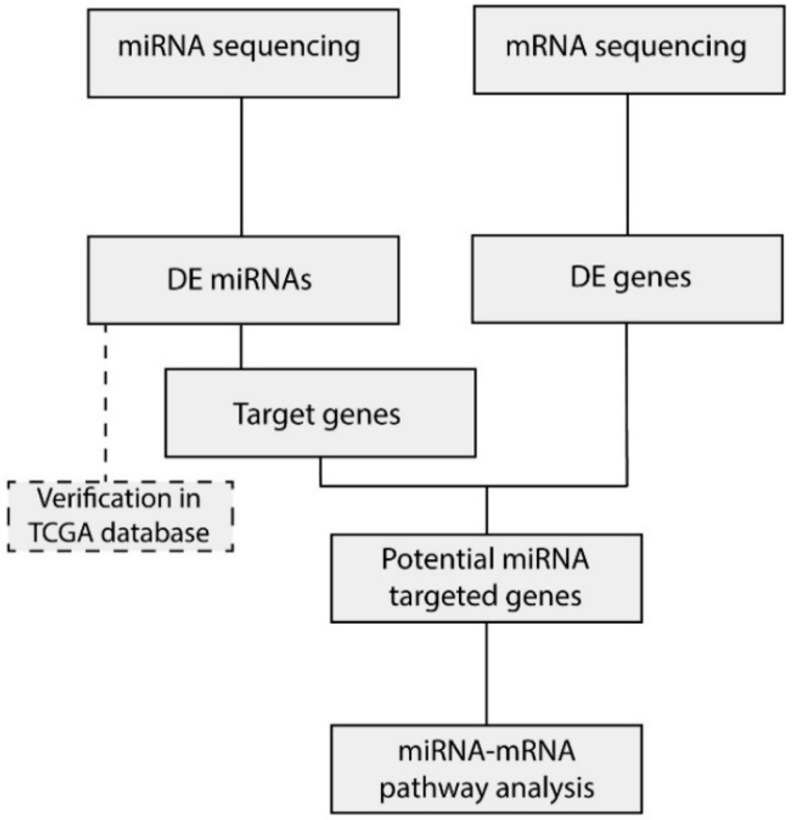
Sample overview and analysis. Flowchart indicating the downstream analysis of the miRNA and mRNA sequencing data. Differentially expressed (DE) miRNAs between the high-risk samples and low/intermediate-risk samples were integrated with the DE genes extracted from the mRNA data. Subsequently, pathway analysis was performed in order to identify which canonical pathways were affected by differential miRNA expression.

Twenty-six UM patients were enrolled in this study and grouped into three subtypes based on risk of developing metastasis (i.e. disease-free survival (DFS) and mutation status). Clinical, molecular, and histopathological characteristics of all patients are listed in Table 1. All patients had a choroidal or ciliary body UM, iris UM were excluded. Seven patients showed a mean DFS of 145 months and had an UM harboring an *EIF1AX* mutation. These patients were included in the low-risk group and did not show disease progression. Twelve patients with a mean DFS of 103 months and an *SF3B1* mutated UM were included in the intermediate-risk group. The high-risk group consisted of seven patients with a mean DFS of 28 months, a *BAP1* mutated tumor, and a negative BAP1 immunohistochemistry (IHC). Within the high-risk UM, all patients died due to metastasis, whereas in the intermediate group three patient were still metastasis-free and died because of other causes. From all 26 samples, mRNA and miRNA sequencing was performed and differentially expressed (DE) miRNA and mRNA were identified (Figure 1). DE-miRNAs were verified in The Cancer Genome Atlas (TCGA) dataset. The target genes identified by the prediction algorithm analysis that also showed a significant negative correlation with the corresponding miRNA were considered to be a potential miRNA target gene and were used subsequently in the expression network.

### 2.2. Identification of Differentially Expressed miRNAs

**Figure 2:**
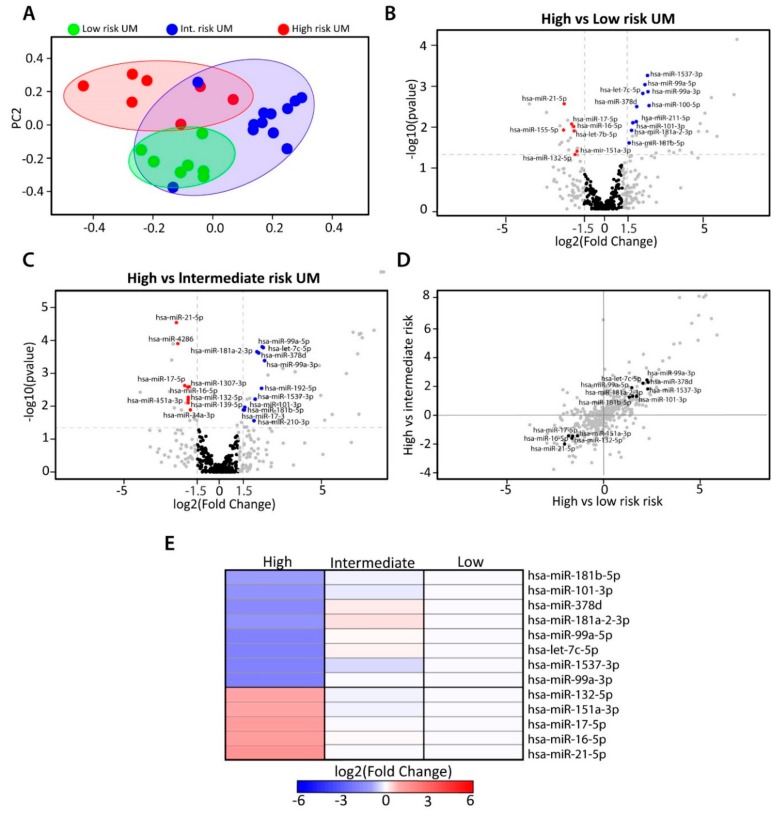
Differential miRNA expression within UM subtypes. (**A**) Principal Component Analysis (PCA) plot showing the unsupervised clustering based on total miRNA expression of all samples. (**B**) Volcano plot indicating which miRNAs are differentially expressed between high- vs. low-risk UM. (**C**) High- vs. intermediate-risk UM. Blue dots indicate downregulation and red dots indicate upregulation of the miRNA (**D**) The correlation between high- vs. low-risk and high- vs. intermediate-risk UM (**E**) Heatmap showing the set of 13 miRNAs identified to be potentially involved in the high-metastatic-risk UM.

To investigate which miRNAs might be involved in the metastatic progression of UM, differential count analyses were performed among the three risk groups. We found 423 mature miRNAs to be expressed in UM. To identify samples with a similar miRNA expression, we generated a principal component analysis (PCA) plot based on total miRNA expression. Unsupervised clustering revealed different clusters; one containing the high-risk UM and the other cluster containing low-risk UM (Figure 2A). The intermediate-risk UM were found in a larger cluster that partially overlapped with the other two clusters. Seventeen miRNAs were differentially expressed between high- vs. low-risk UM, 20 DE-miRNAs were identified between high- vs. intermediate-risk UM, and two DE-miRNAs were found between low- vs. intermediate-risk UM (Figure 2B,C and Figure S1). Since we aimed to identify miRNAs involved in the early metastasis of UM, we continued with thirteen miRNAs that were differentially expressed in the high-risk group, compared to the other two groups (Figure 2D). Of these thirteen, five miRNAs were upregulated significantly in high-risk UM and eight miRNAs were downregulated significantly in high-risk UM. Specifically, miRNA 132-5p, 151a-3p, 17-5p, 16-5p, and 21-5p all had a higher expression in high-risk tumors, whereas miRNA 181b-5p, 101-3p, 378d, 181a-2-3p, 99a-5p, let-7c-5p, 1537-3p, and 99a-3p showed downregulation in the high-risk UM (Figure 2E). The DE-miRNAs were also analyzed in TCGA dataset (Figure S2).

### 2.3. Integration of miRNA and mRNA Expression Data Identifies Target Genes

**Figure 3:**
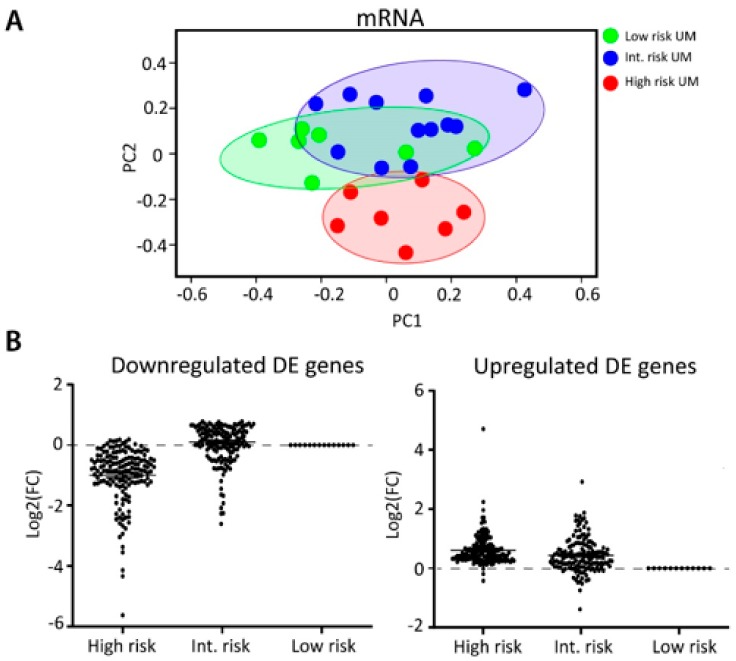
Integration of miRNA and mRNA data. (**A**) PCA plot showing the unsupervised clustering of all samples based on total mRNA expression. (**B**) DE genes were clustered according to gene expression pattern. One group contained all genes that showed downregulation in the high-risk group, compared to the low-risk group. The other group contained genes that were upregulated in the high-risk group.

To explore the biological relevance of the differentially expressed miRNAs involved in the metastatic progression of UM, such as their interaction with cancer-related genes, miRNA expression data were integrated with mRNA expression data. We performed mRNA sequencing on all 26 tumors and unsupervised clustering based on total mRNA expression showed a similar clustering as seen with the miRNA data (Figure 3A). The high-risk UM clustered in a separate group, whereas the low and intermediate-risk UM showed a partial overlap. All DE genes (log2FC > 1.5) with a *p*-value of less than 0.05 were separated into two clusters; one cluster contained all genes that showed downregulation in the high-risk UM and the other group contained the upregulated genes (Figure 3B). From this list, we subsequently generated a target gene list for each miRNA by using four different prediction algorithms (Figure S3). If a gene was predicted to be a target gene by at least two prediction algorithms and showed anti-correlation with its predicted miRNA, the gene was included into our analysis (*n* = 106) (Table 2).

### 2.4. miRNA Target Genes From Several Cancer-Related Pathways

**Figure 4:**
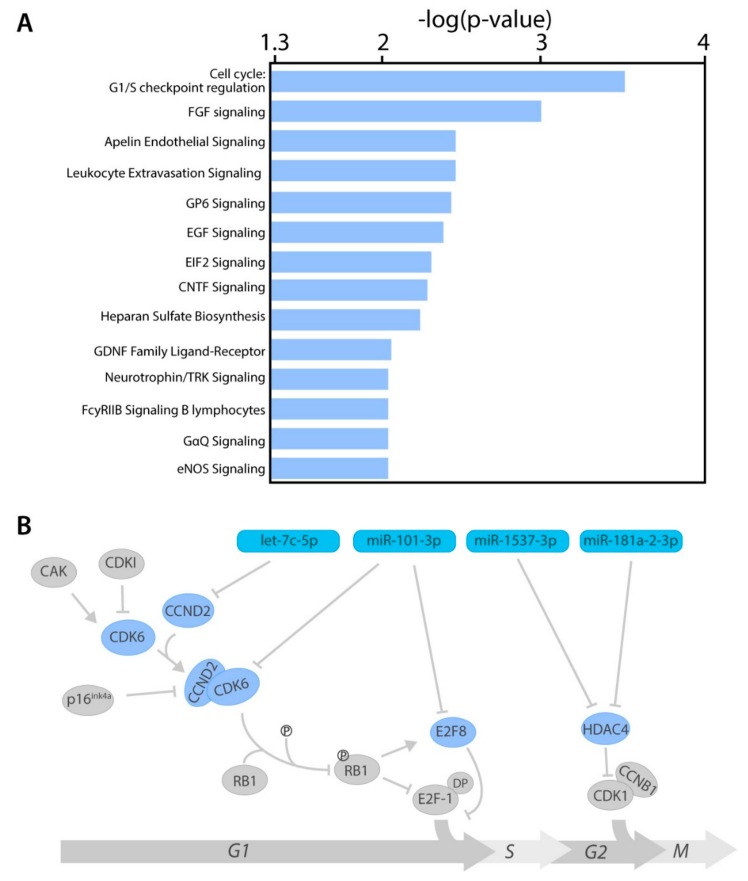
Ingenuity Pathway Analysis. (**A**) Ingenuity pathways with at least three target genes and a log (*p*-value) above 2. (**B**) A cluster analysis visualizing the involvement of DE-miRNAs in the cell cycle. The light blue nodes indicate genes targeted by DE-miRNAs (darker blue nodes), whereas the grey genes are not.

The 106 identified target genes were analyzed by Ingenuity Pathway Analysis (IPA) software, to elucidate which canonical pathways were mainly affected by aberrant miRNA expression (Table S1). One of the most significantly enriched pathways was the cell cycle: G1/S-checkpoint regulation pathway. Moreover, 13 other pathways from IPA also showed a highly significant enrichment, including fibroblast growth factor signaling, Apelin endothelial signaling, and Leukocyte extravasation signaling (Figure 4A). Four target genes were found to be involved in cell cycle regulation (Figure 4B and Figure S4). MiRNA-101-3p inhibits the cyclin-dependent kinase 6 (CDK6) which regulates the G1/S phase transition by inhibiting RB1. It also targets E2F transcription factor 8 (E2F8), which inhibits the G1/S phase transition. The miRNA let-7c-5p inhibits cyclin D2 (CCND2) which binds to CDK6, in order to activate the protein kinase complex. Histone deacetylase 4 (HDAC4) is being targeted by two different miRNAs; miRNA-1537-5p and microRNA-181a-2-3p. Whereas all other genes are involved in the G1/S phase transition, HDAC is mainly active in the G2/M phase transition.

## 3. Discussion

In this study we identified a set of miRNAs that are likely to be involved in the metastatic progression of UM by comparing miRNA expression in high-metastatic-risk UM and low/intermediate-metastatic-risk UM. Hierarchical clustering of total miRNA expression showed three different clusters, which corresponded to metastatic risk. In the UM samples, 423 mature miRNAs were shown to be present, of which 13 miRNAs were differentially expressed between low-, intermediate- or, high-risk UM. MiRNAs that are highly expressed in high-risk UM include several known oncomirs such as miRNA-17-5p [25,26,27], miRNA-151a-3p [28], and miRNA-21-5p [29,30,31,32]. MiRNA-17-5p has been described to promote cell proliferation, invasion, and metastasis through several mechanisms, such as PTEN repression, downregulation of EGFR2, and TGFBR2 targeting. Whereas, miRNA-21-5p and miR-151a-3p have been shown to be involved in the epithelial-mesenchymal transition necessary for metastasis. However, we also identified two new potential oncomirs; miRNA-16-5p and miRNA-132-5p. Interestingly, miRNA-16-5p has been described to be stably expressed in breast cancer and normal breast tissue [33], indicating that aberrant miRNA expression differs per tumor type. We observed a downregulation of eight miRNAs, of which most are known to function as tumor suppressors. MiRNA-99a-5p has been shown to inhibit cell proliferation in bladder and breast cancer [34,35]. Whereas miRNA-101-3p is involved in suppressing epithelial-to-mesenchymal transition, necessary for metastasis [36,37,38].

Several studies have investigated miRNA expression in UM, of which most are done in cell lines or very small sample sizes. These studies already identified miRNAs that were shown to be differentially expressed in high-risk UM, such as miRNA-21, miRNA-146b, miRNA-143, miRNA-199a, and miRNA-134 [17,23,32]. However, not all of these previously identified miRNAs showed differential expression in our dataset. The lack of overlap between these studies and our results can be explained by the employment of different techniques and different tissues (cell lines versus tumor tissue). The majority of the articles describing the miRNA expression in UM analyze the miRNA expression by microarrays, which is known to produce data that does not fully overlap with RNA sequencing data. Comparing our dataset to The Cancer Genome Atlas (TCGA) dataset, which is the only study that performed miRNA analysis in UM by using next-generation sequencing, we did observe an overlap (e.g., miRNA-21-5p, miR-101-3p, miR-181a-2-3p, miR-181b-5p, let-7c-5p, and miRNA-17-5p) [39]. As shown in Figure S2, the observed fold changes of each miRNA have the same directionality in both studies, but the observed fold changes and corresponding *p*-values do show some differences. This could be explained by a platform bias, but also by the fact that we could not differentiate in our own analysis of the TCGA dataset between the 3p- and 5p-miRNAs.

In order to determine the downstream effect of the DE-miRNAs, we integrated miRNA data with matching mRNA data containing expression data of target genes by at least two different prediction algorithms. Since miRNAs are known to downregulate or degrade mRNA of their target genes, we only selected genes that were negatively correlated with miRNA expression. Four of these identified target genes (HDAC4, CDK6, E2F8, and CCND2) play a crucial role in the regulation of the cell cycle. The development of cancer is tightly linked to changes in the activity of the cell cycle [40]. In normal cells there is a checkpoint between the G1 phase and S phase, in order to regulate proliferation. This checkpoint is controlled by several regulators, such as CDK6 [41]. Cancer cells; however, require increased cell division in order to proliferate and invade other tissues and one way to achieve this is by aberrant miRNA expression. Previous research has shown that high metastatic risk UM vastly express Ki-67, a protein that is only present in actively dividing cells, indicating that high-metastatic-risk UM has a greater proliferative activity than low-metastatic-risk UM [42]. Since no UM-specific mutations have been identified in cell cycle-related genes, this indicates that miRNAs probably play a crucial role in cell cycle deregulation in UM. We also observed several target genes to be deregulated in the EIF2 signaling pathway. Protein synthesis is a regulated process in the cell and initiation of translation requires several eukaryotic initiation factors (eIFs), such as eIF2. In cancer cells the function of these eIFs is hampered, inhibiting translation and, thereby, promoting translation of mRNA by alternative mechanisms [43]. Incorrect translation of oncogenes and tumor suppressor genes can promote abnormal proliferation in cancer cells. Restoring these eIFs in UM cells could reduce the oncogenic potential of UM and might; therefore, be an interesting therapeutic target [44].

Another pathway that could affect the metastatic potential of UM cells is epidermal growth factor (EGF) signaling. Several studies show involvement of aberrant EGF signaling in the development of several cancer types [45]. The EGF pathway plays a crucial role in several cellular processes, such as proliferation, migration, and survival. In addition, the fibroblast growth factor (FGF) pathway contributes to the same cellular processes [46]. All of these processes could make uveal melanocytes more malignant and promote metastasis. However, functional assays in which a specific miRNA is overexpressed or knocked-down in an UM cell line are still needed to investigate to what extent these DE-miRNAs contribute to metastasis. Since one miRNA can target a large number of genes and most studies use target genes identified by online prediction algorithms, it is important to perform these additional experiments. For several miRNAs this has already been done in UM cell lines or other cancer cell lines; overexpressing miRNA-21 in UM cell lines resulted in increased migration and invasion [32]. Whereas inhibition of miRNA-17 suppresses the epithelial-mesenchymal transition in gastric cancer cell lines [47].

Because of their stable nature in tissues and body fluids, it is often suggested that miRNAs are excellent biomarkers for clinical applications. They could serve as early prognosis indicators and as a marker for therapy efficiency [48,49]. In our study we have observed a large number of differentially expressed miRNAs between low/intermediate-risk UM and high-risk UM, indicating that these miRNAs could be used to distinguish between these UM subtypes. However, differentiating between low- and intermediate-risk UM based on miRNAs expression will be challenging, since we only identified two miRNAs to be differentially expressed. The miRNA signature specific for high-risk UM might also be detectable in the plasma of UM patients and could; therefore, be a promising non-invasive biomarker to identify high-risk UM patients. This has already been shown in several cancer types, such as germ-cell tumor [50]. Non-invasive biomarkers will allow us to provide all UM patients, including the patients treated with radiotherapy or proton therapy, with a prognosis and good clinical counselling.

This study shows that miRNAs play an important role in the deregulation of several oncogenic pathways in UM and can, thereby, promote metastatic spreading to distant organs, such as the liver. Differentially expressed miRNAs could be an interesting biomarker for metastatic risk in diagnostics, furthermore it also offers us a promising therapeutic target. Until now, no successful treatment has been developed for metastatic UM. miRNA mimics and molecules targeted at miRNAs (anti-miRs) have shown promising results in preclinical development and could compensate for the upregulation of oncogenic pathways, and thereby aid in UM management and treatment [51,52,53].

## 4. Materials and Methods

### 4.1. Tissue Samples

26 patients diagnosed with UM were selected from our Rotterdam Ocular Melanoma Studygroup (ROMS) database. The specimens were collected from enucleated eyes between 1995 and 2010 at the Erasmus University Medical Centre and the Rotterdam Eye Hospital (Rotterdam, The Netherlands). Shortly after surgery, half of the tumor was snap-frozen in liquid nitrogen to allow for DNA and RNA extraction. The other half of the eye was formalin-fixed paraffin-embedded and stained with hematoxylin and eosin for routine histological examination by an ophthalmic pathologist to verify neoplastic nature. This study was performed according to the guidelines of the Declaration of Helsinki (MEC-2009-375, 12 November 2009) and informed consents were obtained at the time of diagnosis.

### 4.2. Mutational Analysis

DNA was extracted from fresh tumor tissue using the QIAmp DNA-mini kit (Qiagen, Hilden, Germany) according to the manufacturer’s instructions. Mutation analysis of *EIF1AX*, *SF3B1* and *BAP1* was carried out using Sanger sequencing as reported in previous publications [4,54] 

### 4.3. Isolation and Sequencing of Small RNA and mRNA

Total RNA was isolated from sections of snap-frozen tumor samples, using the Qiagen miRNeasy isolation kit (Qiagen) according to the manufacturer’s manual. The quantity and purity of the RNA was determined using Bioanalyzer (Agilent Genomics, Santa Clara, CA, USA). A total amount of 4 μg RNA (RIN > 7.0) was used for the preparation of the small RNA and larger RNA libraries using the Ion Total RNA-seq kit (Thermofisher Scientific, Waltham, MA, USA) following the manufacturer’s protocol. Both RNA libraries were subsequently sequenced with the Ion Proton sequencer (Thermofisher Scientific).

### 4.4. Analysis of the Sequencing Data

Adapter sequences, low quality reads, and reads containing poly-N were removed from the generated RNA sequenced data using the Torrent Suite Software V 4.4.3 (Thermofisher Scientific, Waltham, MA, USA). Reads shorter than 15 nucleotides were removed from downstream analysis in the small RNA dataset. The remaining reads were aligned against a miRBase reference genome using an in-house developed script [55]. Read per million mapped reads (RPM) was applied to quantify the expression of each miRNAs. Differential expression and fold changes of miRNAs between each of the patients sets was determined using the statistical package DEseq, with the cut-off FDR < 0.05 (v.1.32.0) [56]. The miRNAs at low expression levels were removed by requiring an average of at least 250 RPM. Short Time-Series Expression Miner [57], under the K-mean clustering method, was used to perform the miRNA expression clustering analysis. The long sequencing reads (>25 bp) were aligned to the human reference genome (hg19) with TopHat2 [58]. Genes at low expression level were removed by the requiring an average of at least 10 RPM. Differentially expressed genes were identified using DEseq [59] with the cut-off fold discovery rate (FDR) < 0.05. Genes were considered to be differentially expressed if they had at least a log2FC of 1.5. The selected resulting genes were used as input for Ingenuity Pathway Analysis (IPA) (Qiagen) for the canonical pathway analysis. All analyses were performed using R statistical environment version 3.5.

### 4.5. miRNA Target Gene Prediction and Validation

To obtain functional information from the differentially expressed miRNAs we integrated mRNA expression data with the miRNA expression data by cluster analysis. A list of putative target genes from the differentially expressed miRNAs was composed using miRwalk 3.0 [60], Targetscan 7.2 [61], Diana micro-T 5.0 [62], and miRDB 6.0 [63]. Genes were considered to be target genes if they were reported by at least two different prediction algorithms. 

### 4.6. Acquisition of TCGA Data

miRNA expression data of 80 UM samples [39] were retrieved from the publicly accessible data repository at the Genomic Data Commons Data Portal (https://portal.gdc.cancer.gov/).

## 5. Conclusions

In this study we elucidated the potential role of miRNAs in the early metastasis of UM by integrating miRNA and mRNA sequencing data derived from 26 UM samples. We showed that differentially expressed miRNAs could play an important role in several oncogenic pathways, such as cell cycle regulation and EGF signaling, which could contribute to the early metastasis of UM. These results do not only bring us one step closer to unraveling the mechanisms that drive UM metastasis, but it also provides us with a promising potential target for future treatment. Targeting these differentially expressed miRNAs could compensate for the deregulation of oncogenic pathways and, thereby, aid in UM treatment.

## Figures and Tables

**Table 1:** The clinical, histological, and molecular characteristics of all 26 uveal melanoma (UM) patients.

Patients Characteristics	Low Risk Group	Intermediate Risk Group	High Risk Group
	(*n* = 7)	(*n* = 12)	(*n* = 7)
**Age**			
Mean ± SD	58 ± 9	50 ± 15	69 ± 14
**Gender, N (%)**			
Male	5 (71)	5 (42)	1 (14)
Female	2 (29)	7 (58)	6 (86)
**Disease free survival**			
Mean ± SD	145.1 ± 45.1	103.3 ± 50.6	28.2 ± 9.26
**Mutation status, N (%)**			
GNAQ	4 (57)	7 (58)	4 (57)
GAN11	3 (43)	5 (42)	3 (43)
EIF1AX	7 (100)	0 (0)	0 (0)
SF3B1	0 (0)	12 (100)	0 (0)
BAP1	0 (0)	0 (0)	7 (100)
**Monosomy 3, N (%)**			
Present	0 (0)	0 (0)	7 (100)
Absent	7 (100)	11 (92)	0 (0)
NE	0(0)	1 (8)	0 (0)
**BAP1 IHC, N (%)**			
Positive	7 (100)	12 (100)	0 (0)
Negative	0 (0)	0 (0)	7 (100)
**Metastasis**			
Present	0 (0)	9 (75)	7 (100)
Absent	7 (0)	3 (25)	0 (0)

**Table 2:** The predicted target genes that show anti-correlation with a specific DE miRNA. An asterisk indicates that more than one miRNA regulates the gene.

miRNA	Target Gene
**let-7c-5p**	ACSL6, AGO4, CACNB4, CCND2, CUX1, ESPL1, FRMD4B, LINGO1, MTDH, PALD1, PARP8, RDX, RGS16 *, RNF217, STARD13
**miR-16-5p**	CNTN3, COL24A1, DIXDC1, ESRRG, EXTL3, FAM110C, FGF2, HS3ST5, ITPR1, MBNL2, OTUD4, PDK4, SLC6A11, SLC7A2, SNRK, SOX5 *, SYT3, VEGFA, ZMAT3
**miR-17-5p**	ARAP2, CDC5L, DCBLD2, ENPP5, ETV1, HMGB3, NR4A3, NTNG1, PCDHA6, SESN3, SLC12A3, TUSC2
**miR-21-5p**	AMER1 *, BCL11A, CSRNP3, IRAK1BP1, LIFR, MEF2C, NKIRAS1, PAIP2B, PCSK6, PDZD2, PRPF4B, STATB1, SCN8A *, SLC22A15, SPRY1, ST6GAL, TIMP3
**miR-99a-5p**	FGFR3, HS3STB1
**miR-99a-3p**	GIMAP1
**miR-101-3p**	ASAP1, ATAD2B, C8orf44-SGK3, CDK6, CLDN11, E2F8, HSD11B2, IMPA1, ITGA8, MAGI2 *, MYCN, PBX3 *, PHACTR2, SALL1, SGK3, SH3PXD2A, SORL1, SRGAP *, STOX2, TRIB1, TSHZ3, ZDHHC21
**miR-132-5p**	ANO10, DUSP7
**miR-151a-3p**	SEC22C
**miR-181a-2-3p**	HDAC4 *, PAG1, RBMS3, ZFP62
**miR-181b-5p**	DERL1, FBXL17, MBOAT2, PLAG, SLC7A, YTHDF3 *
**miR-378d**	PHF21B, PTPN11
**miR-1537-3p**	NSMAF, SNX8, SPC25, TNFSF15

* Targeted by multiple DE miRNAs.

## Supplementary Materials

The following are available online at https://www.mdpi.com/2072-6694/11/6/815/s1. Figure S1: Volcano plot with DE-miRNAs high- vs. intermediate-risk UM, high- vs. low-risk UM, and low- vs. intermediate-risk UM. Figure S2: miRNA expression analysis of TCGA cohort. Figure S3: Overlap in the target genes predicted by four different algorithms (Diana, miRDB, miRWalk, and Targetscan). Figure S4: Heatmap showing the differential expression of the four cell-cycle related genes; CDK6, CCND2, E2F8 and HDAC4. Table S1: IPA target analysis.
