# Supplementary material for: Aberrant MicroRNA Expression and Its Implications for Uveal Melanoma Metastasis
Source: Cancers (Basel). 2019 Jun 12;11(6):815. doi: 10.3390/cancers11060815 (PMC6628189; doi:10.3390/cancers11060815)
Supplement: Supplementary file 1 [file cancers-11-00815-s001.pdf]

Supplementary Materials: Aberrant MicroRNA Expression and Its Implications for Uveal Melanoma Metastasis

Kyra N. Smit, Jiang Chang, Kasper Derks, Jolanda Vaarwater, Tom Brands, Rob M. Verdijk, Erik A.C. Wiemer, Hanneke W. Mensink, Joris Pothof, Annelies de Klein and Emine Kilic

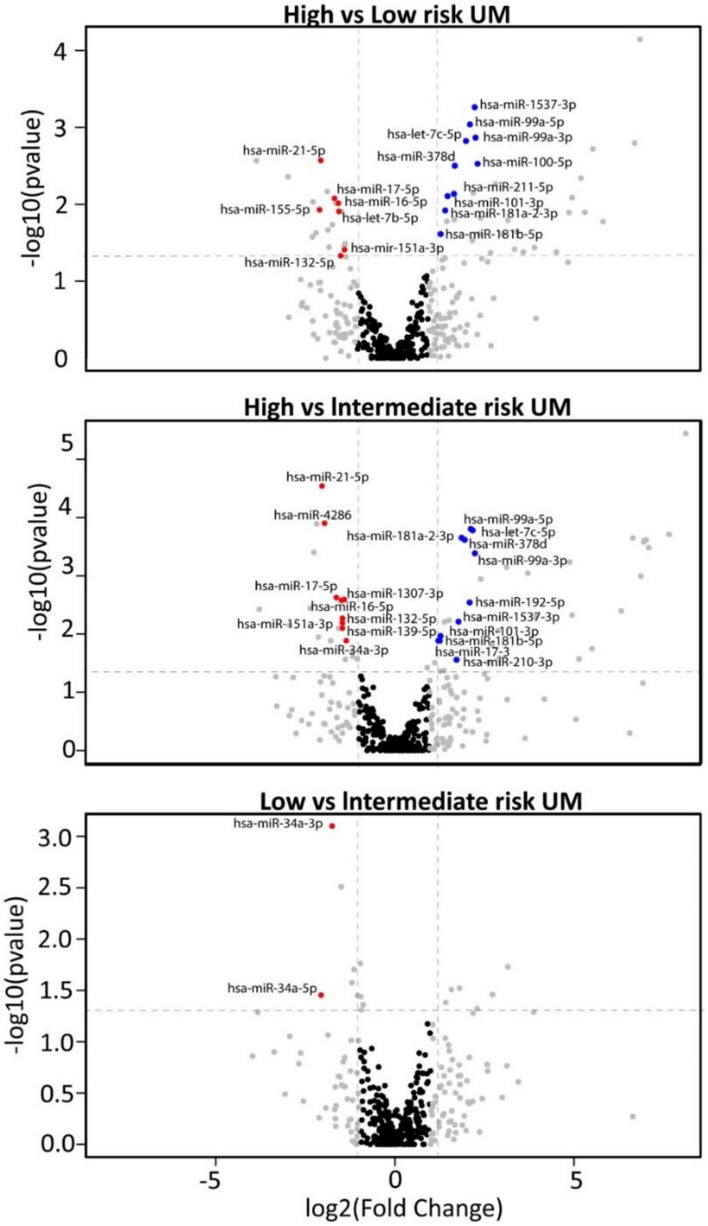

Figure S1. Volcano plots indicating the differentially expressed miRNAs between high vs low-risk UM, high vs intermediate-risk UM and low vs intermediate-risk UM.

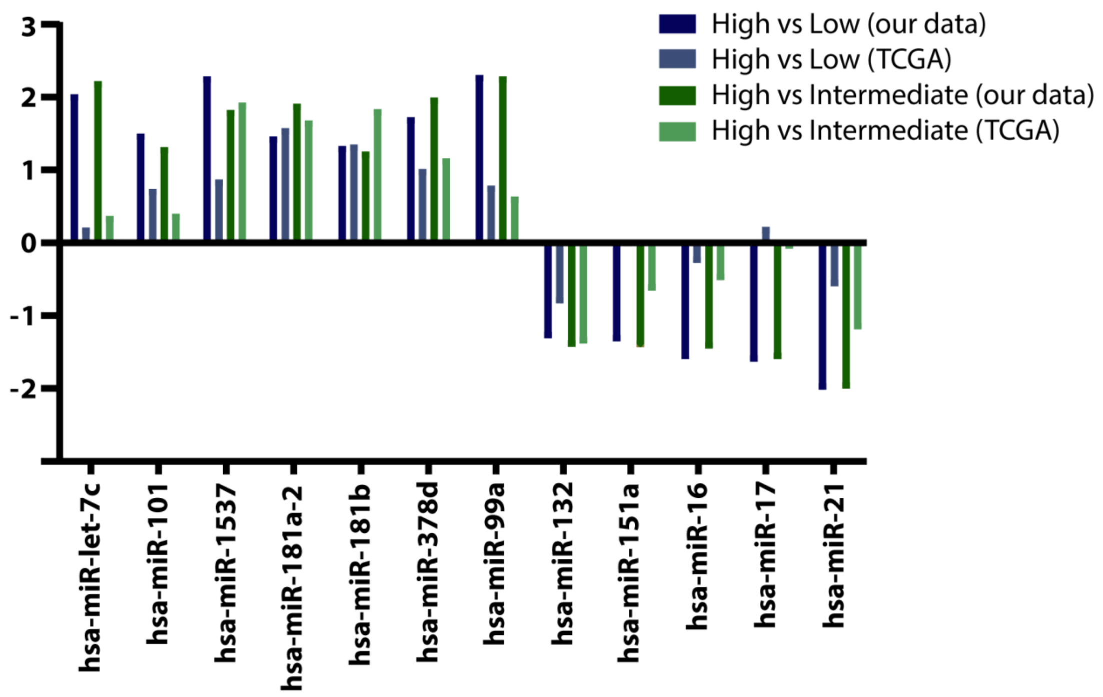

Figure S2. miRNA expression analysis of TCGA cohort.

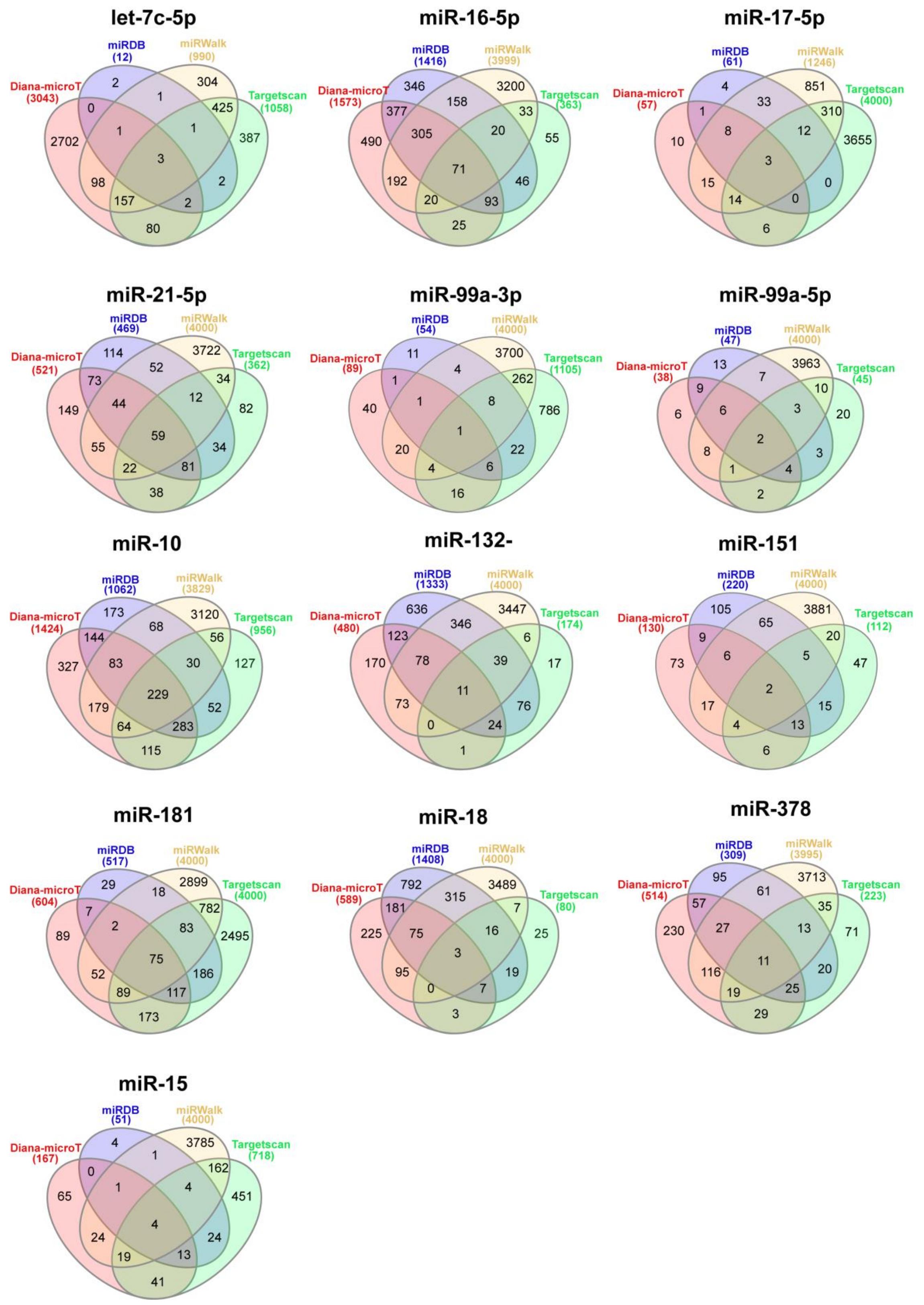

Figure S3. Overlap in the target genes predicted by four different algorithms (Diana, miRDB, miRWalk and Targetscan).

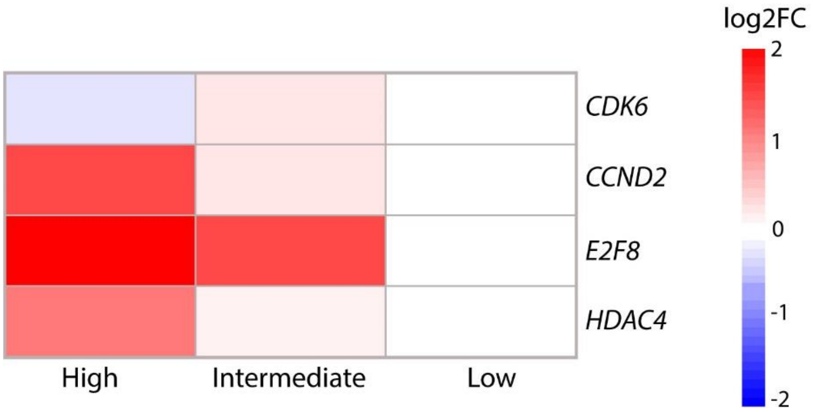

Figure S4. Heatmap showing the differential expression of the four cell-cycle related genes; CDK6, CCND2, E2F8 and HDAC4.

Table S1. IPA target analysis.

Ingenuity Canonical Pathways	−log (*p*-Value)	Molecules
Chronic Myeloid Leukemia Signaling	3,63E00	FGFR3,HDAC4,PTPN11,CDK6,E2F8
Cell Cycle: G1/S Checkpoint Regulation	3,53E00	HDAC4,CCND2,CDK6,E2F8
Osteoarthritis Pathway	3,27E00	FGFR3,VEGFA,TIMP3,HDAC4,FGF2,MEF2C
Cyclins and Cell Cycle Regulation	3,22E00	HDAC4,CCND2,CDK6,E2F8
Role of NFAT in Cardiac Hypertrophy	3,1E00	FGFR3,HDAC4,PTPN11,CACNB4,MEF2C,ITPR1
Non-Small Cell Lung Cancer Signaling	3,08E00	FGFR3,PTPN11,CDK6,ITPR1
Type II Diabetes Mellitus Signaling	3,06E00	FGFR3,PTPN11,CACNB4,ACSL6,NSMAF
FGF Signaling	3,01E00	FGFR3,PTPN11,FGF2,ITPR1
Endocannabinoid Cancer Inhibition Pathway	3,01E00	FGFR3,VEGFA,CCND2,PTPN11,NSMAF
Glioblastoma Multiforme Signaling	2,77E00	FGFR3,PTPN11,CDK6,ITPR1,E2F8
Nitric Oxide Signaling in the Cardiovascular System	2,65E00	FGFR3,VEGFA,PTPN11,ITPR1
Pancreatic Adenocarcinoma Signaling	2,53E00	FGFR3,VEGFA,PTPN11,E2F8
Glioma Signaling	2,52E00	FGFR3,PTPN11,CDK6,E2F8
Apelin Endothelial Signaling Pathway	2,46E00	FGFR3,HDAC4,PTPN11,MEF2C
Leukocyte Extravasation Signaling	2,45E00	FGFR3,TIMP3,CLDN11,PTPN11,RDX
GP6 Signaling Pathway	2,4E00	FGFR3,PTPN11,COL24A1,ITPR1
EGF Signaling	2,34E00	FGFR3,PTPN11,ITPR1
Corticotropin Releasing Hormone Signaling	2,31E00	VEGFA,CACNB4,MEF2C,ITPR1
EIF2 Signaling	2,3E00	FGFR3,VEGFA,MYCN,PTPN11,AGO4
CNTF Signaling	2,28E00	FGFR3,LIFR,PTPN11
Heparan Sulfate Biosynthesis (Late Stages)	2,24E00	HS3ST3B1,EXTL3,HS3ST5
Hereditary Breast Cancer Signaling	2,19E00	FGFR3,HDAC4,PTPN11,CDK6
Heparan Sulfate Biosynthesis	2,14E00	HS3ST3B1,EXTL3,HS3ST5
Small Cell Lung Cancer Signaling	2,08E00	FGFR3,PTPN11,CDK6
GDNF Family Ligand-Receptor Interactions	2,08E00	FGFR3,PTPN11,ITPR1
Neurotrophin/TRK Signaling	2,07E00	FGFR3,PTPN11,SPRY1
FcγRIIB Signaling in B Lymphocytes	2,04E00	FGFR3,PTPN11,CACNB4
Glioma Invasiveness Signaling	2,04E00	FGFR3,TIMP3,PTPN11
Gαq Signaling	2,03E00	FGFR3,PTPN11,RGS16,ITPR1
eNOS Signaling	2,02E00	FGFR3,VEGFA,PTPN11,ITPR1
Cardiac Hypertrophy Signaling (Enhanced)	2,01E00	FGFR3,HDAC4,PTPN11,FGF2,TNFSF15,MEF2C,ITPR1

Renal Cell Carcinoma Signaling	2	FGFR3,VEGFA,PTPN11
Bladder Cancer Signaling	1,95E00	FGFR3,VEGFA,FGF2
HER-2 Signaling in Breast Cancer	1,93E00	FGFR3,PTPN11,CDK6
VEGF Family Ligand-Receptor Interactions	1,93E00	FGFR3,VEGFA,PTPN11
Ceramide Signaling	1,87E00	FGFR3,PTPN11,NSMAF
Role of NFAT in Regulation of the Immune Response	1,84E00	FGFR3,PTPN11,MEF2C,ITPR1
B Cell Receptor Signaling	1,84E00	FGFR3,PTPN11,PAG1,MEF2C
Xenobiotic Metabolism Signaling	1,83E00	FGFR3,HS3ST3B1,HDAC4,PTPN11,HS3ST5
FAK Signaling	1,81E00	FGFR3,PTPN11,ASAP1
Amyotrophic Lateral Sclerosis Signaling	1,78E00	FGFR3,VEGFA,PTPN11
p53 Signaling	1,78E00	FGFR3,CCND2,PTPN11
Calcium Signaling	1,77E00	HDAC4,CACNB4,MEF2C,ITPR1
UVA-Induced MAPK Signaling	1,77E00	FGFR3,PTPN11,PARP8
VEGF Signaling	1,77E00	FGFR3,VEGFA,PTPN11
Clathrin-mediated Endocytosis Signaling	1,76E00	FGFR3,VEGFA,PTPN11,FGF2
Apelin Cardiomyocyte Signaling Pathway	1,76E00	FGFR3,PTPN11,ITPR1
Neuropathic Pain Signaling In Dorsal Horn Neurons	1,74E00	FGFR3,PTPN11,ITPR1
Mouse Embryonic Stem Cell Pluripotency	1,74E00	FGFR3,LIFR,PTPN11
IL-8 Signaling	1,71E00	FGFR3,VEGFA,CCND2,PTPN11
Breast Cancer Regulation by Stathmin1	1,71E00	FGFR3,PTPN11,ITPR1,E2F8
Role of p14/p19ARF in Tumor Suppression	1,71E00	FGFR3,PTPN11
T Cell Receptor Signaling	1,71E00	FGFR3,PTPN11,PAG1
Synaptogenesis Signaling Pathway	1,7E00	FGFR3,PTPN11,SYT3,CACNB4,ITPR1
Dermatan Sulfate Biosynthesis (Late Stages)	1,69E00	HS3ST3B1,HS3ST5
Telomerase Signaling	1,69E00	FGFR3,HDAC4,PTPN11
IL-9 Signaling	1,68E00	FGFR3,PTPN11
Paxillin Signaling	1,67E00	FGFR3,PTPN11,ITGA8
CREB Signaling in Neurons	1,67E00	FGFR3,PTPN11,CACNB4,ITPR1
iCOS-iCOSL Signaling in T Helper Cells	1,66E00	FGFR3,PTPN11,ITPR1
Chondroitin Sulfate Biosynthesis (Late Stages)	1,64E00	HS3ST3B1,HS3ST5
Myo-inositol Biosynthesis	1,63E00	IMPA1
HIF1α Signaling	1,63E00	FGFR3,VEGFA,PTPN11
Integrin Signaling	1,62E00	FGFR3,PTPN11,ASAP1,ITGA8
fMLP Signaling in Neutrophils	1,6E00	FGFR3,PTPN11,ITPR1
Actin Cytoskeleton Signaling	1,59E00	FGFR3,PTPN11,FGF2,RDX
Renin-Angiotensin Signaling	1,59E00	FGFR3,PTPN11,ITPR1
Role of NANOG in Mammalian Embryonic Stem Cell Pluripotency	1,59E00	FGFR3,LIFR,PTPN11
CD28 Signaling in T Helper Cells	1,58E00	FGFR3,PTPN11,ITPR1
Adipogenesis pathway	1,58E00	FGFR3,HDAC4,FGF2
Role of Tissue Factor in Cancer	1,58E00	FGFR3,VEGFA,PTPN11
STAT3 Pathway	1,58E00	FGFR3,VEGFA,FGF2
Docosahexaenoic Acid (DHA) Signaling	1,56E00	FGFR3,PTPN11
IL-6 Signaling	1,54E00	FGFR3,VEGFA,PTPN11
CCR3 Signaling in Eosinophils	1,53E00	FGFR3,PTPN11,ITPR1
Chondroitin Sulfate Biosynthesis	1,52E00	HS3ST3B1,HS3ST5
Dermatan Sulfate Biosynthesis	1,49E00	HS3ST3B1,HS3ST5
Apelin Pancreas Signaling Pathway	1,49E00	FGFR3,PTPN11
Huntington's Disease Signaling	1,49E00	FGFR3,HDAC4,PTPN11,ITPR1

Axonal Guidance Signaling	1,49E00	FGFR3,VEGFA,NTNG1,PTPN11,SRGAP1,<br>LINGO1
Gα12/13 Signaling	1,49E00	FGFR3,PTPN11,MEF2C
Human Embryonic Stem Cell Pluripotency	1,48E00	FGFR3,PTPN11,FGF2
IL-23 Signaling Pathway	1,48E00	FGFR3,PTPN11
Ephrin A Signaling	1,45E00	FGFR3,PTPN11
Ovarian Cancer Signaling	1,42E00	FGFR3,VEGFA,PTPN11
Pyridoxal 5'-phosphate Salvage Pathway	1,42E00	PRPF4B,CDK6
Melanoma Signaling	1,4E00	FGFR3,PTPN11
UVB-Induced MAPK Signaling	1,38E00	FGFR3,PTPN11
Lymphotoxin β Receptor Signaling	1,37E00	FGFR3,PTPN11
Relaxin Signaling	1,37E00	FGFR3,VEGFA,PTPN11
Molecular Mechanisms of Cancer	1,37E00	FGFR3,CCND2,PTPN11,CDK6,E2F8
Role of IL-17A in Arthritis	1,36E00	FGFR3,PTPN11
ERK5 Signaling	1,34E00	PTPN11,MEF2C
Regulation of eIF4 and p70S6K Signaling	1,34E00	FGFR3,PTPN11,AGO4
Role of Pattern Recognition Receptors in Recognition of Bacteria and Viruses	1,33E00	FGFR3,PTPN11,TNFSF15
PKCθ Signaling in T Lymphocytes	1,32E00	FGFR3,PTPN11,CACNB4
MSP-RON Signaling Pathway	1,32E00	FGFR3,PTPN11
Thrombopoietin Signaling	1,3E00	FGFR3,PTPN11
Endometrial Cancer Signaling	1,3E00	FGFR3,PTPN11
IL-2 Signaling	1,3E00	FGFR3,PTPN11
Aldosterone Signaling in Epithelial Cells	1,3E00	FGFR3,PTPN11,ITPR1
